# Antimicrobial Efficiency of Hypochlorous Acid and Its Effect on Some Properties of Alginate Impression Material

**DOI:** 10.1155/2023/8584875

**Published:** 2023-11-14

**Authors:** Bayan S. Khalaf, Shorouq M. Abass, Aseel Mohammed Al-Khafaji, Moamin I. Issa

**Affiliations:** Department of Prosthodontics, College of Dentistry, University of Baghdad, Baghdad 1417, Iraq

## Abstract

Dental clinicians and professionals need an affordable, nontoxic, and effective disinfectant against infectious microorganisms when dealing with the contaminated dental impressions. This study evaluated the efficiency of hypochlorous acid (HOCl) as an antimicrobial disinfectant by spraying technique for the alginate impression materials, compared with sodium hypochlorite, and its effect on dimensional stability and reproduction of details. HOCl with a concentration of 200 ppm for 5 and 10 min was compared with the control group (no treatment) as a negative control and with sodium hypochlorite (% 0.5) as a positive control. *Candida albicans*, *Staphylococcus aureus*, and *Pseudomonas aeruginosa* were selected to assess the antimicrobial activity with the colony forming unit test in addition to the dimensional stability and reproduction of details tests. The results revealed that HOCl had significant antimicrobial activity against all tested microorganisms and experimental time. Interestingly, HOCl showed no impact on the dimensional stability of alginate impression material. HOCl could be an effective antimicrobial agent for alginate impression material without interfering with their surface details and dimensional stability.

## 1. Introduction

All surfaces of impressions need to be disinfected with a hospital‑grade disinfectant. Despite studies in the literature, there is still a need for further research and development on the interaction between disinfectant solutions and impression materials, optimal exposure time, ideal concentration of chemical disinfectant, and ideal composition [1, 2]. There is a risk of cross‑contamination in the dental clinics and laboratories by contaminated dental impressions with human blood and saliva; therefore, professionals should follow coordinated disinfection protocols [3].

Dentists commonly use alcohol, chlorine combination, aldehydes, biguanides, iodide combinations, phenols, and ammonium as chemical disinfectants [4]. These disinfectants are generally used for immersion or spraying to disinfect dental materials [5]. A considerable amount of literature has been published on the different disinfectants, disinfection procedures, products, and contact times. However, a universally recognized disinfection is still lacking.

Most dental clinicians and laboratories do not consider chemical disinfectants' effect on the dental impressions. In addition, most available impression materials were not formulated for disinfection procedures, and logically these chemical solutions may affect the different properties of impressions, including the dimensional stability and the production of a detailed gypsum model. Thus, many studies investigated the effect of the disinfection method on the dimensional stability and surface properties of impression materials [6, 7]. Immersion of the impressions is a more effective disinfection technique than with spray [8].

However, the immersion technique can cause dimensional changes in the impressions, decreasing the quality of prosthetic results achieved in dental practices [9].

The various impression materials may react with the chemical disinfectants according to the method, type of disinfectant, concentration, and even duration. The percentage of (0.5%) sodium hypochlorite was advised for disinfection of irreversible hydrocolloid (alginate) impressions. Spray disinfection was preferred over prolonged immersion due to the imbibition-related deterioration of the immersion technique [10]. However, sodium hypochlorite has several disadvantages due to its toxicity, eye and skin irritation, corrosiveness, and daily preparation [11].

One of the most effective chemicals used is hypochlorous acid (HOCl) which is natural and nontoxic [10]. There are many uses for HOCl as a disinfectant including dentistry, disinfection of wounds, and as virucidal as well as antimicrobial agent [12, 13]. A variety of viruses could be inactivated with HOCl in less than 60 s, including coronaviruses [14]. A concentration of 200 ppm for 1 min was efficient in disinfecting surfaces with the noroviruses and other enteric viruses. Even when diluted to 20 ppm, HOCL successfully disinfected surfaces with the viral contaminants within 10 min of contact time [12].

To the best of the authors' knowledge, no other studies were found in the literature investigating the effects of hypochlorous acid as a disinfectant for alginate impression materials. Thus, the present study evaluated the antimicrobial efficiency of hypochlorous acid as a disinfectant by spraying technique for alginate impression materials compared with sodium hypochlorite. Dimensional stability and reproduction of the alginate impression materials' details were also investigated when disinfection.

## 2. Materials and Methods

The specimens prepared for this study were from alginate impression material (Tropicalgin, Zhermack, Italy) for the different tests; antimicrobial, dimensional stability, and reproduction of details. These specimens were distributed into four test groups; HOCL (200 ppm) for 5 and 10 min, sodium hypochlorite (% 0.5) (Microvem, Turkey), and sterile distal water as a control group as shown in Table 1.

### 2.1. Antimicrobial Efficiency Test

The specimens for the antimicrobial efficiency test were prepared by mixing the alginate powder with sterile distilled water for 45 s using a mixing spatula in a rubber bowl according to the manufacturers' instructions. Then, the alginate mix was immediately placed in a modified 5-cc sterile plastic hypodermic syringe with an internal diameter of 12 mm until setting of the material. Slices of 2-mm thickness were made using a surgical blade number 11 from the end of the syringe after extruding 2 mm of the set alginate material [15]. All of the specimens were later sterilized in an autoclave at 121°C and 15 psi [16].

The three tested microorganisms were isolated from patients who attended the teaching hospital of the College of Dentistry of the University of Baghdad in Baghdad, Iraq and according to the ethical approval of the ethical committee with reference number: 661, project number 661222 on September 3, 2022. A standard inoculum of bacteria was used for each type of susceptibility test for the bacterial suspension and the yeast preparation. The preparation of the standard inoculums achieved a matching of turbidity of 1.5 × 10^8^ colony forming unit, CFU/ml, equivalent to 0.5 McFarland. *Candida albicans* was isolated and identified using gram stain, germ tube, and API candida tests. *Staphylococcus aureus* was identified using polymerized chain reaction (PCR), while *Pseudomonas aeruginosa* was identified using real-time quantitative PCR (qPCR). The Mueller–Hinton broth was used in the cultivation and incubation for 24 hr of all microorganisms [17].

The antimicrobial test consisted of four groups as control and for the different disinfection methods. Each group consisted of three subgroups of three specimens for each type of microorganism used in this study. The alginate impression specimens were placed in the microbial suspension test tubes and vortex-mixed for 60 s. After removal from the microbial suspension, the specimens were disinfected with 200-ppm HOCL for two intervals of 5 or 10 min or 0.5% sodium hypochlorite for 10 min by 10 puffs sprayed within 15 s. The specimens of the control group were excluded from the disinfection process. The specimens were washed with water and then placed in the test tube of 10-ml distilled water and vortex-mixed for 60 s. Then, the tenfold serial dilution method was used by taking 100 *μ*l of the distilled water from the test tubes and inoculating it on blood agar plates. Later the agar plates were incubated aerobically for 48 hr at 37°C [18].

The specimens were washed with water and then placed in a test tube of 10-ml distilled water and vortex-mixed for 60 s. Next, a tenfold serial dilution method was used, which involves taking 100 *μ*l of distilled water from test tubes, inoculating it onto blood agar plates, and then aerobic incubation for 48 hr at 37°C [18]. The number of colonies (CFU/ml) on the agar plates was counted after incubation and according to the following equation [19]:

(1) $$\begin{array}{c} \text{Numbers of CFU}/\text{ml}=\left(\text{Colony number}\times \text{Dilution Factor}\right)/\text{Volume of the culture plate.} \end{array}$$

### 2.2. Dimensional Stability and Reproduction of Details Test

**Figure 1:**
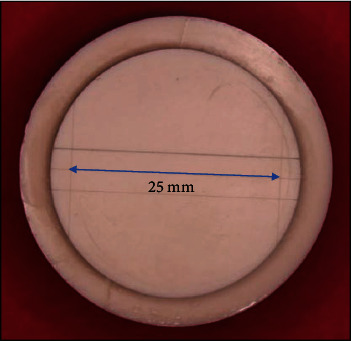
Ruled test block used for the dimensional stability and reproduction of details test.

The ruled test block used for the dimensional stability and reproduction of details test was according to ISO specification number 1563:1990 for alginate impression material [20]. This test block had three lines, 25 mm long, engraved on the surface and two parallel cross-lines that crossed perpendicularly to the three lines previously mentioned. The two cross-lines were separated by a distance of 25 mm as shown in Figure 1. The ruled test block was cleaned with alcohol and air dried before placing a ring around it as a mold during making the alginate impression specimens. The alginate impression material was mixed according to the manufacturer's instructions, placed in the ring surrounding the ruled test block, and covered with a glass slab loaded with 1-kg weight for 3 min before removal from the mold.

**Figure 2:**
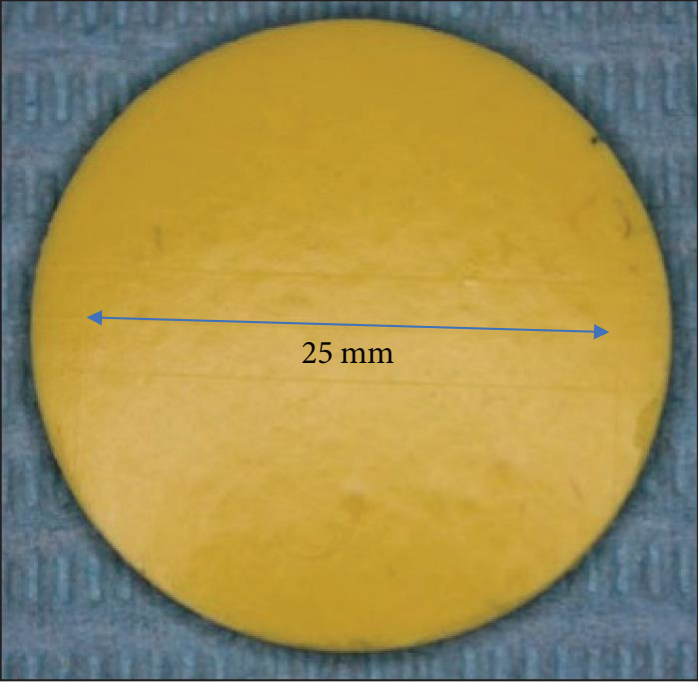
Alginate impression material specimen used for the dimensional stability and reproduction of details test.

The number of alginate samples was 40 and evenly distributed among four test groups as shown in Figure 2. The first group (control) was sprayed with distilled water and stored for 10 min in a closed container. The second and third test groups were disinfection with HOCL for 5 and 10 min, respectively. The fourth test group was disinfection with NaOCl for 10 min [21]. The disinfectant was left on the surface of the specimens in a closed container for 5 or 10 min according to the test group requirements [22]. Finally, distilled water was used to wash the specimens thoroughly for 1 min before air drying and storage in a closed container until testing.

The reproduction of details was by observation immediately after separation under low-angle illumination without magnification. The reproduction of details was considered satisfactory if the 50 *μ*m wide line was continuous for the full length of 25 mm between cross-lines in at least two of the three specimens prepared.

The dimensional stability was evaluated by measuring the distance between the two cross-lines in an image of the alginate impression specimen's surface using Corel DRAW X3 Version 13 [23, 24]. The image was obtained from digitization using a digital camera (Canon EOS 1200D) with a fixed distance and a ruler adjacent to the specimens for calibration of the length, as suggested by Oliveira et al. [25]. Calculation of the dimensional change was used according to the following formula:

(2) $$\begin{array}{c} \text{Dimensional change (\%)}=\left(A-B\right)/A\times 100. \end{array}$$

Reading (*A*) was the distance separating the cross-lines on the ruled test block (25 mm). While, reading (*B*) was the distance separating the cross-lines on the alginate impression specimens.

All data were entered into SPSS version 15 program and analyzed with ANOVA and Tukey HSD as the post hoc test with a significance level of *P* < 0.05.

## 3. Results

### 3.1. Antimicrobial Efficiency Test

**Figure 3:**
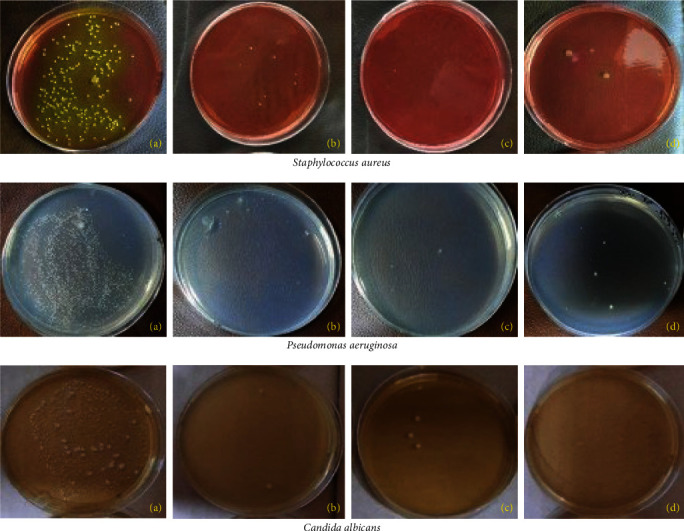
Antimicrobial efficiency test for different microorganisms (*Staphylococcus aureus*, *Pseudomonas aeruginosa*, and *Candida albicans*): (a) Control group, (b) HOCL acid for 5 min, (c) HOCL acid for 10 min, and (d) NaOCL group.

Means of the CFU count of the specimens disinfected with the different solutions (hypochlorous acid and sodium hypochlorite) and control group for the different microorganisms (*C. albicans*, *S. aureus*, and *P. aeruginosa*) are presented in Table 2 and Figure 3. The results showed a reduction in CFU count for all the test groups except for the control group which had the highest mean.

The one-way ANOVA analysis of CFU showed a significant difference between all the test groups for all three types of microorganisms (*C. albicans*, *S. aureus*, and *P. aeruginosa*) (*P* value <0.05), as shown in Table 3.

Analysis with the Tukey HSD post hoc test of the mean of CFU between the control and the test groups for each type of microorganism showed a significant difference (*P* < 0.05), as shown in Table 3. Comparison between the NaOCl and the HOCl test groups for 5 and 10 min was insignificant (*P* value >0.05), as shown in Table 4.

### 3.2. Dimensional Stability and Reproduction of Details Test

Table 5 presents the results of the dimensional change. The one-way ANOVA analysis showed that the difference was insignificant between all the test groups (*P* value >0.05), as shown in Table 6.

The reproduction of details test showed complete reproduction of the 50 *µ*m line, which was continuous, sharp, and well-defined for the entire distance between cross-lines in all alginate specimens for all test groups (100% for all 10 specimens of the 4 test groups).

## 4. Discussion

The method of spray disinfection was adapted in this study because of its wide use and the absence of the disadvantages associated with using the immersion technique, which may include the adverse effects of dimensional stability [26].

In this research NaOCl at 0.5% concentration was selected as a positive control since it was recommended for use as a disinfectant agent for alginate impression materials [27]. Basmaci et al. [10] suggested disinfecting the alginate impression materials with 0.5% sodium hypochlorite. Also, they stated that disinfecting the alginate impression materials by spraying is recommended, as prolonged immersion could cause imbibition-related deterioration [10].

HOCL gained popularity recently due to the worldwide outbreak of the COVID-19, which generated concern from the World Health Organization (WHO). HOCl, in an aqueous solution, could act as a powerful oxidizing agent by dissociation into H^+^ and OCl^−^ and aggregating and denaturing proteins [28]. In addition, HOCl inactivates viruses by forming nitrogen-centered radicals and chloramines and, consequently, results in breaks in single- and double-stranded DNA of microoganisims [14, 29].

Egusa et al. [30] showed that hazardous microorganisms like *S. aureus*, *C. albicans*, and *P. aeruginosa* could be isolated from alginate impressions of the patient's arches. These pathogens are opportunistic and have the ability to spread throughout the oral cavity [30, 31]. Thus, *S. aureus*, *C. albicans*, and *P. aeruginosa* were selected to evaluate the disinfection's efficacy in this study.

Results of the current study confirmed that the HOCl acid and the NaOCl disinfection effectively reduced the CFU of tested microorganisms. The findings of Badrian et al. [16], Correia-Sousa et al. [32], and Hardan et al. [33] all agreed with the results of this study. As an oxidizing agent, hypochlorite is fungicidal, bactericidal, and sporicidal, and the active part is the hypochlorous acid [34]. This hypochlorous acid solution is potent by reacting with the structural proteins, like capsid or surface compounds, lipid envelop, and nucleic acids (DNA or RNA) of viruses [35].

The results of this study presented no change in the dimensions with the use of HOCl acid and NaOCl disinfectant agent when compared with the control group, as observed by Hamedi et al. [36]. This result may relate to the spray technique used in the study which had no adverse effects on the dimensional changes.

The results of this study showed that the use of HOCI as a disinfectant agent had no impact on the reproducibility of details which may be related to the purification method and short duration.

The limitation of this study was that the compatibility with gypsum products and the surface hardness of the resultant cast need to be investigated after disinfection with HOCL and can generally be recommended. This study may prove the practical clinical efficacy of using HOCL to the disinfect alginate impression materials.

## 5. Conclusion

The influence of hypochlorous acid as an antimicrobial disinfectant on the dimensional stability and reproduction of details of alginate impression materials was evaluated compared to the untreated and sodium hypochlorite-treated groups. The results showed that hypochlorous acid and sodium hypochlorite spray disinfectants were able to disinfect the alginate impression material with an effective reduction in microorganism CFU count of *C. albicans*, *S. aureus*, and *P. aeruginosa*. Both hypochlorous acid and sodium hypochlorite showed no remarkable impact was found on the dimensional stability and reproduction of details of the alginate impression material. Therefore, treatment with such antimicrobial, nontoxic, and inexpensive materials could be promising for reducing contamination of alginate impressions without affecting the surface details and dimensional stability.

## Figures and Tables

**Table 1:** Experimental groups of alginate impression material used in this study.

Test groups	Treatment
Control	No treatment
NaOCL	Spray disinfection with NaOCl for 10 min
HOCL 5	Spray disinfection with HOCL for 5 min
HOCL 10	Spray disinfection with HOCL for 10 min

**Table 2:** Descriptive statistics of colony forming unit (CFU) counts/ml × 10^6^ for the different disinfectant solutions for *Candida albicans*, *Staphylococcus aureus*, and *Pseudomonas aeruginosa*.

	Disinfectant	Minimum	Maximum	Mean	Std. deviation
*Candida albicans*	Control	25	25	25	0.00000
	NaOCl	0.0	0.3	0.1	0.14142
	HOCl (5 min)	0.0	0.0	0.0	0.00000
	HOCl (10 min)	0.1	0.5	0.2	0.17889

*Staphylococcus aureus*	Control	25	25	25	0.00000
	NaOCl	0.0	0.5	0.16	0.23022
	HOCl (5 min)	0.0	0.7	0.32	0.32711
	HOCl (10 min)	0.2	0.3	0.24	0.05477

*Pseudomonas aeruginosa*	Control	25	25	25	0.00000
	NaOCl	0.0	1.4	0.72	0.49699
	HOCl (5 min)	0.8	1.2	1.0	0.14142
	HOCl (10 min)	0.3	1.4	0.82	0.49699

**Table 3:** Statistical analysis by one-way ANOVA of the means of CFU between the different test groups for all three types of microorganisms (*Candida albicans*, *Staphylococcus aureus*, and *Pseudomonas aeruginosa*).

Microorganism		Sum of squares	*df*	Mean square	*F*	Sig.
*Candida albicans*	Between groups	2,323.914	3	774.638	59587.538	.000
	Within groups	0.208	16	.013		
	Total	2,324.122	19			

*Staphylococcus aureus*	Between groups	2,299.030	3	766.343	18805.971	.000
	Within groups	0.652	16	.041		
	Total	2,299.682	19			

*Pseudomonas aeruginosa*	Between groups	2,187.890	3	729.297	5675.459	.000
	Within groups	2.056	16	.129		
	Total	2,189.946	19			

**Table 4:** Post hoc tests analysis (Tukey HSD) of colony forming unit (CFU) counts of different disinfectant solutions for each microorganism (*Candida albicans*, *Staphylococcus aureus*, and *Pseudomonas aeruginosa*).

Microorganism	Test groups	Mean difference	Std. error	95% Confidence interval		Sig.
				Lower bound	Upper bound	
*Candida albicans*	Control-NaOCl	24.90000^*∗*^	0.07211	24.6937	25.1063	0.000
	Control-HOCl (5 min)	25.00000^*∗*^	0.07211	24.7937	25.2063	0.000
	Control-HOCl (10 min)	24.78000^*∗*^	0.07211	24.5737	24.9863	0.000
	NaOCl-HOCl (5 min)	0.10000	0.07211	−0.1063	0.3063	0.525
	NaOCl-HOCl (10 min)	−.12000	0.07211	−0.3263	0.0863	0.373

*Staphylococcus aureus*	Control-NaOCl	24.84000^*∗*^	0.12767	24.4747	25.2053	0.000
	Control-HOCl (5 min)	24.68000 ^*∗*^	0.12767	24.3147	25.0453	0.000
	Control-HOCl (10 min)	24.76000^*∗*^	0.12767	24.3947	25.1253	0.000
	NaOCl-HOCl (5 min)	−.16000	0.12767	−0.5253	0.2053	0.604
	NaOCl-HOCl (10 min)	−.08000	0.12767	−0.4453	0.2853	0.922

*Pseudomonas aeruginosa*	Control-NaOCl	24.28000^*∗*^	0.22672	23.6314	24.9286	0.000
	Control-HOCl (5 min)	24.00000^*∗*^	0.22672	23.3514	24.6486	0.000
	Control-HOCl (10 min)	24.18000^*∗*^	0.22672	23.5314	24.8286	0.000
	NaOCl-HOCl (5 min)	−0.28000	0.22672	−0.9286	0.3686	0.615
	NaOCl-HOCl (10 min)	−0.10000	0.22672	−0.7486	0.5486	0.970

^*∗*^Significant difference.

**Table 5:** Descriptive statistics of dimensional stability for the specimens disinfected with the different disinfection solutions.

Disinfectant	Minimum	Maximum	Mean	Std. deviation
Control	25.00	27.00	26.0000	1.05409
NaOCl	25.00	26.00	25.7000	0.48305
HOCl (5 min)	25.00	26.00	25.2000	0.42164
HOCl (10 min)	25.00	27.00	26.0000	1.05409

**Table 6:** Comparison of dimensional stability for the specimens disinfected with the different disinfectant solutions by one-way ANOVA.

	Sum of squares	*df*	Mean square	*F*	Sig.
Between groups	4.275	3	1.425	2.165	0.109
Within groups	23.700	36	0.658		
Total	27.975	39			

## Data Availability

The data that support the findings of this study are available from the corresponding author upon reasonable request.
